# SNPs Array Karyotyping Reveals a Novel Recurrent 20p13 Amplification in Primary Myelofibrosis

**DOI:** 10.1371/journal.pone.0027560

**Published:** 2011-11-14

**Authors:** Giuseppe Visani, Maria Rosaria Sapienza, Alessandro Isidori, Claudio Tripodo, Maria Antonella Laginestra, Simona Righi, Carlo A. Sagramoso Sacchetti, Anna Gazzola, Claudia Mannu, Maura Rossi, Michele De Nictolis, Massimo Valentini, Meris Donati, Roberto Emiliani, Francesco Alesiani, Stefania Paolini, Carlo Finelli, Stefano A. Pileri, Pier Paolo Piccaluga

**Affiliations:** 1 Hematology and Hematopoietic Stem Cell Transplant Center, San Salvatore Hospital, Pesaro, Italy; 2 Molecular Pathology Laboratory, Department of Hematology and Oncology “L. e A. Seràgnoli”, Hematopathology and Hematology Sections, University of Bologna, S. Orsola-Malpighi Hospital, Bologna, Italy; 3 Department of Human Pathology, University of Palermo, Palermo, Italy; 4 Department of Pathology, San Salvatore Hospital, Pesaro, Italy; 5 Clinical Pathology Laboratory, San Salvatore Hospital, Pesaro, Italy; 6 Oncohematology, Bartolomeo Eustachio Hospital, San Severino Marche, Italy; University of Sao Paulo – USP, Brazil

## Abstract

The molecular pathogenesis of primary mielofibrosis (PMF) is still largely unknown. Recently, single-nucleotide polymorphism arrays (SNP-A) allowed for genome-wide profiling of copy-number alterations and acquired uniparental disomy (aUPD) at high-resolution. In this study we analyzed 20 PMF patients using the Genome-Wide Human SNP Array 6.0 in order to identify novel recurrent genomic abnormalities. We observed a complex karyotype in all cases, detecting all the previously reported lesions (del(5q), del(20q), del(13q), +8, aUPD at 9p24 and abnormalities on chromosome 1). In addition, we identified several novel cryptic lesions. In particular, we found a recurrent alteration involving cytoband 20p13 in 55% of patients. We defined a minimal affected region (MAR), an amplification of 9,911 base-pair (bp) overlapping the *SIRPB1* gene locus. Noteworthy, by extending the analysis to the adjacent areas, the cytoband was overall affected in 95% of cases. Remarkably, these results were confirmed by real-time PCR and validated *in silico* in a large independent series of myeloproliferative diseases. Finally, by immunohistochemistry we found that SIRPB1 was over-expressed in the bone marrow of PMF patients carrying 20p13 amplification. In conclusion, we identified a novel highly recurrent genomic lesion in PMF patients, which definitely warrant further functional and clinical characterization.

## Introduction

Primary myelofibrosis (PMF) is a chronic myeloproliferative neoplasm (MPN) in which the clonal transformation of the hematopoietic stem cell (HSC) results in the prominent expansion of megakaryocytes and granulocytes in the bone marrow (BM) [1]. In its full-blown stage, PMF is associated with the progressive deposition of extracellular matrix components in the BM parenchyma configuring the picture of myelofibrosis (MF), which results from the persistent stimulation of BM mesenchymal components by myeloid clone-derived growth factors and cytokines [2], [3]. Such a perturbation of the BM homeostasis may be profound in the advanced phases of the disease and eventually results in the occurrence of extramedullary haematopoiesis consequent to a defective HSC BM niche [1]. Similar dynamics of BM stroma disarrangement may sustain the development of MF during the natural course of MPNs other than PMF, such as polycythemia vera, and these cases, accounting for around 25% of MF cases, are accordingly defined secondary myelofibrosis (SMF) [4].

The molecular pathogenesis of PMF is largely unknown, though a number of genomic abnormalities have been associated to this disease. In particular, different point mutations involving Janus kinase 2 (*JAK2*), Myeloproliferative Leukemia Virus (*MPL*), *TET* oncogene family member 2 (*TET2*), Sex Combs-Like 1 (*ASXL1*), Casitas B-lineage lymphoma proto-oncogene (*CBL*), and *IKAROS* family zinc finger 1 (*IKZF1*) have been recently described in MPNs and also in PMF [5]. Of note, mutations occurring at *JAK2* locus on 9p24 chromosome appear to play a pivotal role in PMF and currently represent a major criterion for diagnosis according to the World Health Organization [1]. However, none of these mutations is specific for PMF being recorded in different MPNs. Interestingly, they were found to be not mutually exclusive nor could be traced back to a common ancestral clone, highlighting a certain degree of heterogeneity in MF genetics. To this regard, the intriguing possibility of independently emerging multiple abnormal clones (that is, leading to oligoclonal rather than monoclonal myeloproliferation) has been even recently raised [5].

At chromosomal level, metaphase cytogenetics (MC) detects an abnormal karyotype of PMF in about 34% of cases at diagnosis, the most frequent lesions being del(20q), del(13q) and abnormalities of chromosome 1 [6]. Additional observed alterations included +8, +9, abnormalities of chromosomes 3, −5/del(5q), −7/del7(q), del(12p) and +21. However there's a wide heterogeneity among patients and specific recurrent patterns have not been identified so far [7]. However, it should be noted that the resolution of conventional karyotyping, or G-banding, is only 3–20 Mb. Conversely, new DNA array–based methods, such as SNP arrays (SNP-A), increased the resolution up to the base-pair (bp) scale, enabling studies of copy number variants (CNVs) and acquired Uniparental Disomy (aUPD). CNVs are amplified or deleted regions ranging in size from intermediate (1–50 kb) to large (50 kb–3 Mb) [8]–[10] and are recognized as a major source of human genome variability. Importantly, change in copy number (CN) is involved in cancer formation and can increase during tumor progression, influencing phenotypes and prognosis [11]–[13]. aUPDs are regions of loss of heterozygosity (LOH) that occurs without concurrent changes in the gene copy number, phenomenon also reported as copy-neutral LOH. These defects are attributed to errors of mitotic recombination in somatic cells and are being increasingly recognized in a variety of neoplasms [14], [15]. Importantly, high-resolution SNP-A can be easily applied in karyotype analysis representing the possible starting point in discovery several pathogenetically relevant alterations. In fact, it does not depend upon the availability of live, dividing cells, and consequently can yield results when routine MC is not informative. Moreover, due to its higher resolution as compared with MC, smaller, otherwise uncovered deletions and duplications can be visualised.

In the field of MPN, SNP-A profiling studies recently highlighted the impact of this new methodology for detecting regions of aUPD, identifying commonly altered regions and suggesting novel target genes [13], [16], [17]. However, all these studies have been conducted on cohorts of patients affected by MPNs in general and no study has so far focused on PMF.

In this study we performed high resolution karyotyping of a cohort of 20 patients affected by PMF using the most recent Affymetrix SNP-A (Genome-Wide Human SNP Array 6.0). Specifically, we studied DNA from tumour samples and normal matched DNA from the same PMF patients, as well as DNA from healthy individuals aiming to identify novel cryptic aberrations.

## Methods

### Patients

We studied 20 PMF patients diagnosed according to WHO criteria [1]. For each patient peripheral blood (PB) sample, bone marrow (BM) aspirate and BM trephine biopsy were collected at diagnosis. Due to the scant material obtained from BM aspirates (often resulting in a dry tap), SNP-A karyotyping was carried out on PB samples. Conversely, conventional metaphase cytogenetic analysis was performed on bone marrow in the only two cases in which an adequate amount material was collected.

The cases were equally divided into a training set (10 PMF) and a test set (10 PMF). The main clinical features were homogeneously represented in the two groups (Table S1). In particular, the patients of the training test and test set reported a median age of 68 and 74 years, a female/male ratio of 50% and 40%, a median follow up of 28,5 and 38,5 months and a leukemic transformation of 0% and 2%, respectively. *JAK2* mutational status was assessed in all cases as previously described [18] (for details see File S1). Ten patients (6 in the training and 4 in the test set) presented with *JAK2* mutations.

In both training and test set the genomic DNA was isolated from peripheral blood myeloid cells. Additionally, in the training set we also studied non neoplastic T-lymphocytes isolated from PB (see below). Moreover, genomic DNA collected from 10 healthy individuals served as further control.

All the study was approved by the local ethical committee (Department Seràgnoli and San Salvatore Hospital Review Boards) and written permission and informed consent have been obtained from all patients before sample collection.

### Purification of myeloid and CD3^+^ cells from peripheral blood

Peripheral blood mononuclear cells (PBMC) and granulocytes were obtained from 20 patients by Ficoll-Paque by density gradient separation (GE Healthcare Wisconsin, USA). Subsequently, in 10 cases (training set), we collected granulocytes, representing the clonal population, on one hand, and the PBMC on the other. The latter were then processed in order to isolate *bona fide* non neoplastic T-lymphocytes. CD3^+^ cells isolation was performed by immunomagnetic labelling of CD3^+^ cells followed by a separation process using the MACS device (Milteniy Biotech, Bergisch Gladbach, Germany). In brief, up to 2×10^8^ PBMNC were washed with PBS and 1% human albumin to reduce the labeling volume to 10 ml. The cells were then incubated with a clinical grade anti-CD3 monoclonal antibody (MoAb) conjugated to microbeads (MACS CD3 reagent, Myiltenyi Biotech) for 20 minutes. After washing, CD3^+^cells were purified on a MS column (Miltenyi Biotech). After selection, cells present in the positive (CD3) and negative (PBMNC) fractions were counted and submitted to flow cytometry analysis. Assessment of CD3^+^ cells before and after the separation process was carried out by labeling of the target cells with anti-CD3-phycoerythrin (PE) MoAb (Becton Dickinson, San Jose, CA, USA), followed by cytometric reanalysis of purified cells performed on a gated population set on scatter properties by FACScan equipment (Becton Dickinson). A minimum of 10,000 events was collected in list mode on FACScan software.

In the other 10 cases (test set), conversely, only granulocytes were collected and used for the subsequent analyses (test set).

Notably, to ensure the collection of adequate neoplastic components, a morphological evaluation of the granulocytic fractions was performed on smears obtained after cytospin enrichment. Actually, the granulocytic fraction usually included a variable proportion of immature myeloid cells, consistently with the diagnosis of PMF. Only samples for which at least 80% of the cellular population was constituted by myeloid elements were selected for the following analysis.

### SNP Array assay

Genomic DNA was extracted using QIAamp DNA Mini Kit (QIAGEN Inc., Valencia,. CA, U.S.A.) Concentration and quality samples were quantified by Nanodrop ND-1000 spectrophotometer running software version 3.0.1 (NanoDrop Technologies, Inc., Rockland DE). DNA was then treated as far manufacturer's instructions (Affymetrix, Santa Clara, CA, USA) and finally hybridized to the Genome-Wide SNP array 6.0 (Affymetrix, Santa Clara, CA, USA).This array contains 906,600 Single Nucleotide Polymorphism (SNP) probes and more than 945,826 copy number variant (CNV) probes, providing marker spacing in the range of as low as 700 bases. Briefly, 500 ng of highly purified genomic DNA from each patient was processed in each step by a provided Affymetrix® Genome-Wide Human SNP Nsp/Sty Assay Kit 5.0/6. and other prescribed kits. First DNA was cut by restriction enzymes NspI and StyI (Nsp I Enzyme, Sty I Enzyme by New England Biolabs) followed by adaptor ligation (T4 DNA Ligase by New England Biolabs). A generic primer that recognizes the adaptor sequence was used to amplify adaptor-ligated DNA fragments (TITANIUM DNA Amplification Kit by Clontech). PCR conditions have been optimized to preferentially amplify fragments in the 200 to 1,100 bp size range. PCR products were then purified (Magnetic Beads by Agencourt) fragmented and labeled with a fluorochrome. After washing, the arrays were incubated with a streptavidin-phycoerythrin conjugate to enable detection of hybridized fragments on the array. The fragment binding (which reflects the genotype) was then measured by a scanner picking up the fluorescent signals on the array feature scanned (GeneChip® 3000 Scanner with 7G upgrade Affymetrix, Santa Clara, CA, USA).

### Data Analysis

Data were analysed using the Affymetrix Genotyping Console (GTC) 4.0 and Partek Genomic Suite 6.5. First, we uploaded on GTC 4.0 all sample attribute (ARR) and intensity data (CEL) files. All the arrays passed the recommended Quality Control (QC) threshold (≥0.4) according to manual instruction (GTC 4.0 manual, Affymetrix, Santa Clara, CA, USA). Thus, we generated CHP files by performing genotyping analysis, based on Birdseed v.2 algorithm and using default parameters.

Subsequently, all CEL and CHP files were uploaded on Partek Genomic suite 6.5. We updated all the arrays to the new human genome build Genome Reference Consortium GRCh37 (ucsc hg19), corrected the GC waviness and used only the latest builds of databases (Refseq, Database of Genomic Variants).

We then performed a paired analysis of the 10 samples, constituting the training set, for which matched normal DNA was available. In particular, we detected amplifications and deletions using Partek genomic segmentation algorithm (10 minimum genomic markers and a p-value threshold ≤0.001.). Subsequently, we performed an unpaired analysis of the 10 cases, constituting the test set. Specifically, we compared the tumor DNA to an homemade reference baseline (made up by DNA from 10 healthy individuals). The latter was preferred to Partek baseline, basing on its consistency with the paired training test results (for details see file S1).

In all copy number analyses we excluded from the study the chromosomes X, Y, the mitochondrion, all the regions with low density markers and finally the CNV segment <1 Kb and UPDs regions <1 Mb. After that, in order to evaluate the prevalence of CNVs within each chromosome we normalized the number of alterations on chromosome length (in Mb).

For further details on SNP-A analysis see file S1.

### TaqMan Copy Number assay

To validate target genomic copy number amplification found by SNP-A analysis, we used a TaqMan Copy Number assay (Applied Biosystems; Foster City, CA). In particular, the target copy number was assessed with a specific TaqMan Copy Number Assay (Hs04039030_cn; Applied Biosystems; Foster City, CA) and TaqMan Copy Number Reference Assay *RNase P* (Applied Biosystems). For each sample 4 repeats of 20 ng of DNA were run in a duplex real-time Polymerase Chain Reaction with TaqMan Universal Genotyping Master Mix, a FAM® dye-labeled TaqMan® Copy Number Assay and a VIC® dye-labeled TaqMan® Copy Number Reference Assay (Applied Biosystems; Foster City, CA). The *RNaseP* reference genes are known to be present in two copies in a diploid genome, regardless of the copy number of the target of interest, and are used to normalize sample input and minimize the variation between the target of the test and reference assay.

The reactions were performed on the genomic DNA from the original tumour samples and, according to the manufacturer's protocol, were run on an ABI 7900HT real-time PCR machine (Applied Biosystems; Foster City, CA) and primarily analyzed by SDS Software 2.3.

The relative quantitation analysis, without calibrator sample, was then performed by CopyCaller™ Software v1.0

### *In silico* validation

To confirm our results in a validation set, we examined two data sets of Affymetrix CEL files publicly accessible in the GEO database (http://www.ncbi.nlm.nih.gov/geo/). Since there were no available series composed exclusively of PMF cases, all the available MPN cases were included in the validation set. In particular, we analysed raw data from 7 patients with PMF blast phase”originally studied by GeneChip® Human Mapping 250K Nsp, and from 39 MPN patients previously analysed by Affymetrix Human Mapping 50K Array Xba 240 (GSE19647) [13]. Moreover, included the raw data from 14 patients affected by MDS/MPN, originally analysed by using our same array (Genome-Wide SNP array 6.0) (GSE21991) [19].

### Immunohistochemical validation

In order to evaluate the expression of SIRPB1 protein, we studied by immunohistochemistry selected cases carrying or not the amplification within the 20p13 cytoband (containing the *SIRPB1* gene locus). Immunohistochemistry was performed as previously reported, on bone marrow trephine biopsies obtained from the posterior iliac crest [3]. Briefly, Four-micrometers-thick sections of bone marrow biopsies were deparaffinized and rehydrated to water, subsequently the slides were microwave treated in Tris-HCl/EDTA buffer pH 9.0 (DakoCytomation) for a total of 20 minutes prior to PBS washing. After neutralization of the endogenous peroxidase with H_2_O_2_ for 10 minutes, the sections were first incubated with protein block (Novocastra, UK) for 10 minutes. Sections were then incubated with the primary polyclonal antibody rabbit anti-human SIRPB1 (Proteintech Group, USA; dilution 1∶50). Incubation time was overnight at 4°C. Normal rabbit serum was used instead of primary antibodies as negative control. Binding of the primary antibody was revealed by a polymer detection system (Novolink max polymer detection system, Novocastra,UK) using DAB (3,3′-Diaminobenzidine, Novocastra,UK) substrate-chromogen. After counterstaining with hematoxylin (Novocastra,UK), the sections were analysed under a Leica DM2000 optical microscope (Leica, Germany) and captions were collected using a Leica DFC320 digital camera (Leica).

## Results

### Identification of CNV, LOH and aUPD

**Figure 1:**
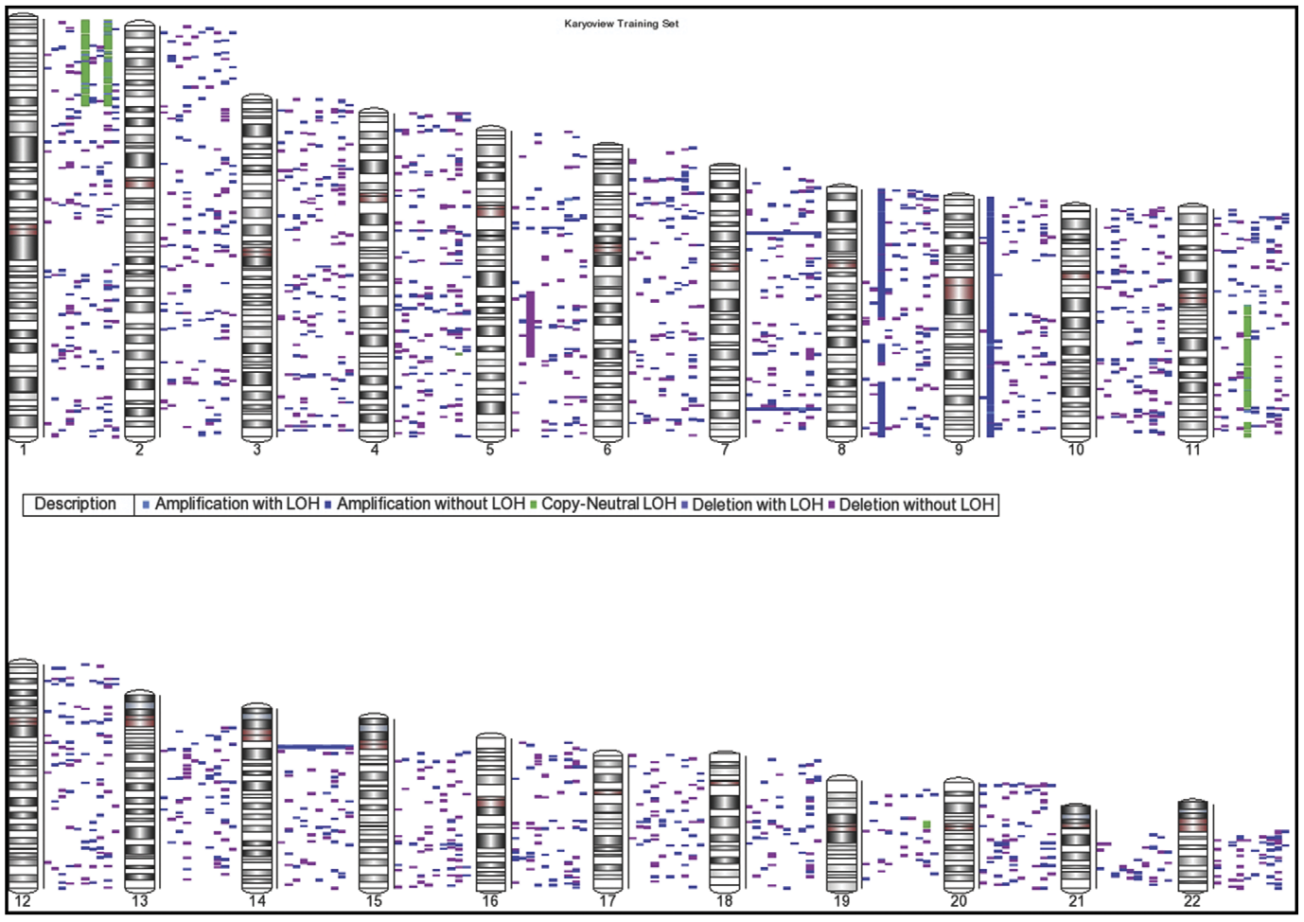
Karyoview of training set. Amplification with LOH (Light blue), Amplification without LOH (Dark blue), Copy Neutral LOH (aUPD, Green), Deletion with LOH (Violet), and Deletion without LOH (Cyclamen) are represented. All the 2,765 lesions recorded in the training set are depicted.

**Figure 2:**
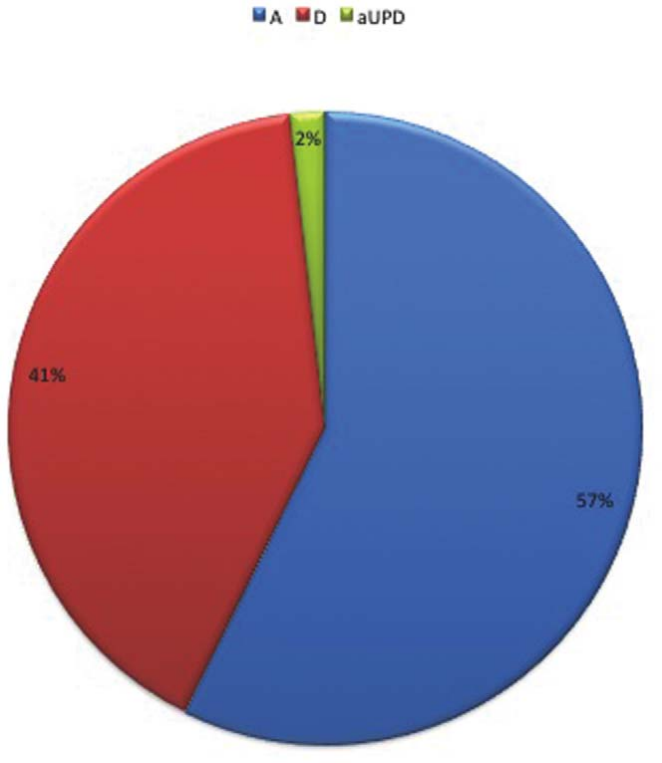
Global distribution of CNVs across PMF genomes. All the CNVs with relative frequencies are reported: amplifications (both with and without LOH) in blue, acquired uniparental disomy regions (aUPD) in green and deletions (both with and without LOH) in red.

First, we aimed to detect unbalanced chromosomal defects as well as aUPD. To address this issue we analysed cases included in the training set (10 cases), and performed a paired analysis for CN determination by genome wide SNP array 6.0. Specifically, we directly compared normal (T lymphocytes) and neoplastic (myeloid cells) matched samples. We identified a total of 2,765 CNVs, ranging in size from 1 kb to 23 Mb (Figure 1) (File S1). In particular, the CNVs were represented by amplifications (N = 1,584, median size 14,5 Kb - range of 1 Kb–2,3 Mb), deletions (N = 1,132, median size 16,7 Kb - range of 1 kb–9 Mb), and aUPD (N = 49, median size 1,9 Mb - range of 1 Mb–7,2 Mb)(Figure 2). Based on the most updated Genomic Toronto Database version (http://projects.tcag.ca/variation/), 1,773 CNVs (64%) of detected CNVs were then regarded as known, while 992 CNVs (36%) were considered as novel.

**Figure 3:**
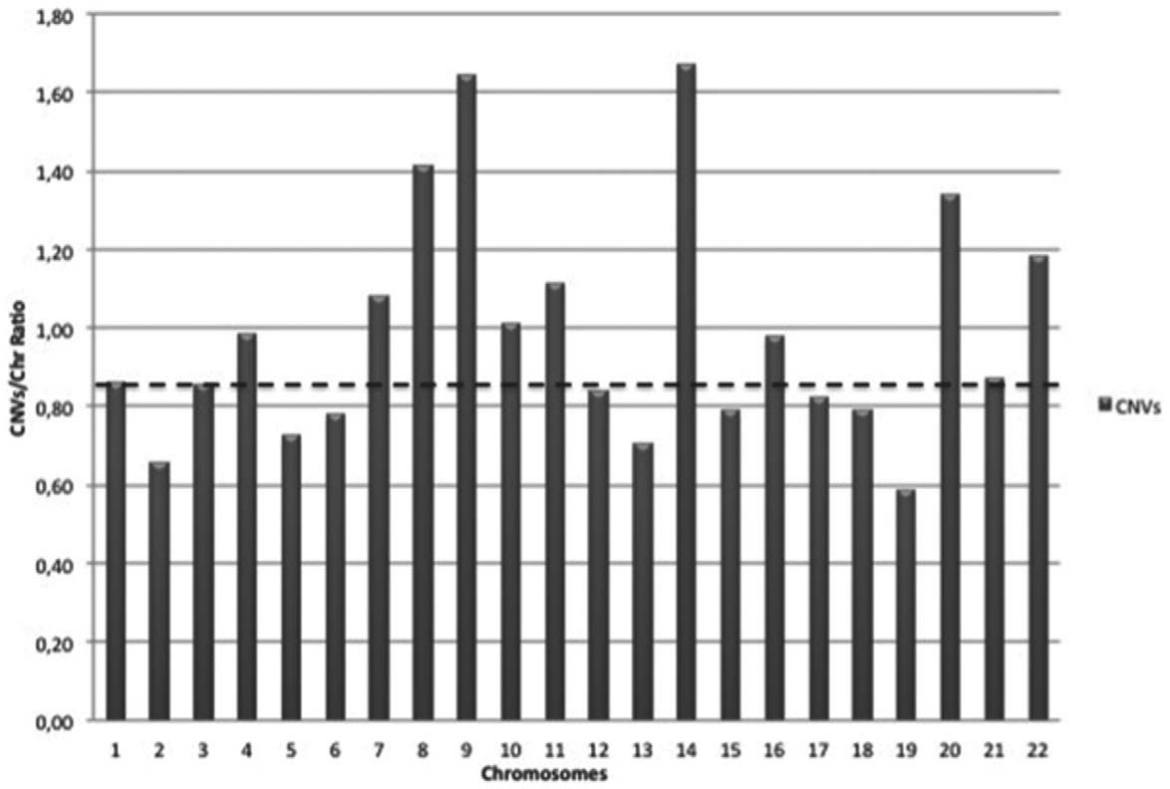
CNVs distribution across chromosomes. On the x axis all the chromosomes except x, y, and mitochondrion are presented. On the y axis the number of CNVs affecting each chromosome after opportune normalization on chromosome size, is reported. The dotted line represents the median value of CNV per chromosome.

We then looked at the CNVs distribution across the chromosomes in order to assess whether specific chromosomes were predominantly affected. After opportune normalization on chromosome size, we observed that the number of CNVs was over the median value in the chromosomes 4, 7, 8, 9, 10, 11, 14, 16, 20 and 22, while it was below in the remaining ones. However the difference was not significant (χ^2^, pvalue = 0,99)(Figure 3).

As far as the single cases were concerned, we identified a mean of 343 CNVs/patient (range 188–602), with a mean size of 168,623 kb and a median size of 15,912 kb. Amplifications were confirmed to be the commonest lesions among patients, though all the types of abnormalities were detected in most instances (Figure S1).

Subsequently, we investigated whether the above CNVs were present in the test set (N = 10 cases) as well. To do this, we first compared the 10 cases of the test set with the DNA obtained from the healthy donors and identified 419 cytobands that were altered. Subsequently, we compared them with the 744 cytobands found to be affected in the training set. Indeed, a significant overlap was recorded between the two groups; specifically, in the test set we confirmed 327/744 lesions (44%)(p = 0.0053)(Figure S2A–B).

**Figure 4:**
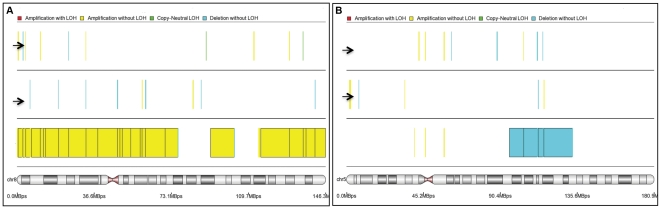
Correspondence between metaphase cytogenetic and SNPs-A karyotyping. A) Trisomy of chromosome 8 identified in one patient (yellow bar) known to carry the same abnormality basing on metaphase cytogenetic results. Please note two different cases randomly selected and presented as negative controls (arrows). B) Deletion of chromosome 5q identified in one patient (light blue bar) known to carry the same abnormality basing on metaphase cytogenetic results. Please note two different cases randomly selected and presented as negative controls (arrows).

Importantly, as internal control, we confirmed the presence of the two abnormalities previously detected by MC: a trisomy 8 and a 5q deletion (Figure 4A–B).

We investigated whether we could identify the lesions detected in the previously published SNP-A profiling studies, which had been performed at a lower resolution. Actually, we identified the presence of different regions of aUPD on chr9 at *JAK2* locus, on chr 13q, on chr 1 and on chr 11. More specifically, concerning the *JAK2* locus, we found 5 amplifications, 1 deletion and 1 aUPD in cases carrying *JAK2* somatic mutations (as detected by PCR and direct sequencing), while 1 amplification, 1 deletion and 1 aUPD were recorded in unmutated cases. In addition, we detected in 2/20 patients and in 3/20 patients respectively, two large region of aUPDs in correspondence to the *MPL* and *CBL* genes. Further validation by direct sequencing was not possible due to the lack of residual material. (Figure S3A-B-C).

### PMF presents with recurrent abnormalities on 20p13

We then looked for CNVs consistently recurrent in our dataset. First the analysis was carried out in the training set (10 cases). As the matched control was represented by T-lymphocytes, we excluded CNVs on chromosomes 7p34, 7p14.1, 14q11.2 (recurrent in all instances) because of possible artefacts due to the physiological alteration secondary to T-cell receptor editing process.

No lesion was found to be present in up to 6/10 cases. Conversely, we found 1 CNV in 5/10 samples, 5 in 4/10 samples, 47 in 3/10 and 285 in at least 2/10 (Figure S4).

Specifically, the most common recurrent lesion was an amplification occurring in 1p31.1 cytoband. However, as this was an already known lesion involving the chromosome 1 [20], we focused on CNVs occurring in 4/10 cases and evaluated them in the test set. These genomic imbalances, in particular, involved the cytobands 1p31.1, 10q23.31, 4q25, 4q31.3 and 20p13. By looking for these lesions in the test set, we found the 20p13 amplification to be the most frequently occurring one (7/10 cases). Such amplification within the 20p13 region was thus overall recorded in 11 out of 20 cases (55%). Subsequently, due to the high prevalence of the lesion and the known involvement of chromosome 20 in myeloid malignancies [21]–[23], we further extended our focus to the entire 20p13 cytoband. In particular, 9/10 patients in the training set showed different alterations affecting the cytoband 20p13. We then tested the presence of 20p13 abnormalities in the test set too, and we found it in 10/10 cases. Thus, overall, 19/20 patients showed abnormalities on chromosome 20p13.

Finally, in order to assess the prevalence of 20p13 abnormalities in an independent panel of cases, we tested an *in silico* validation set including 14 cases of MDS/MPN, 7 cases of PMF in blast phase and 39 previously-reported MPN cases for which high SNP karyotiping CEL files were available (http://www.ncbi.nml.nih.gov/projects/geo/) [13], [19]. We could confirm the presence of lesions affecting the 20p13 cytoband in 7/14 MDS/MPN patients, 5/7 PMF blast phase patients, and in 1/39 MPN patient (Figure S5).

**Figure 5:**
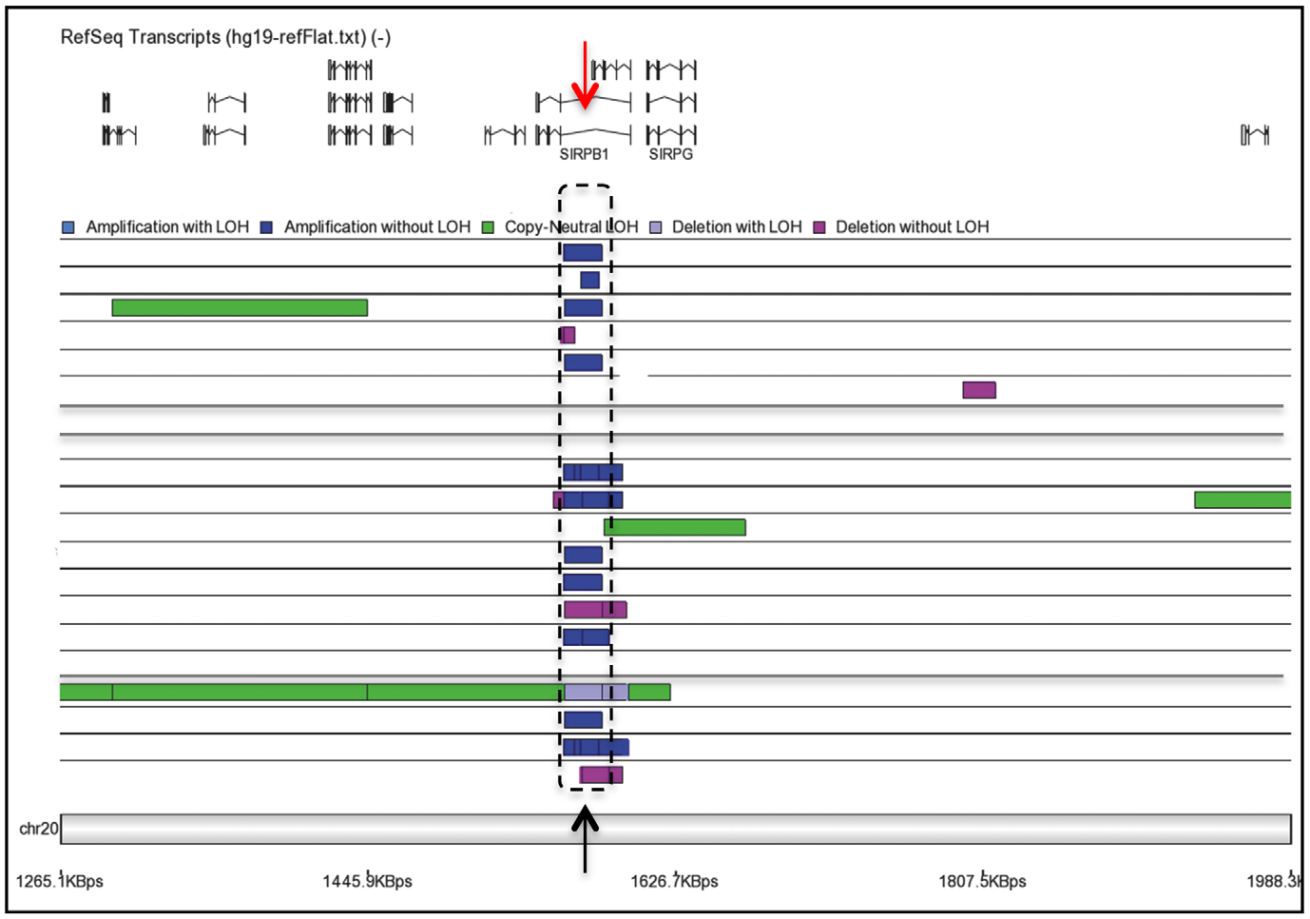
Minimally affected region (MAR) definition. Genomes from all patients are represented as single rows. MAR (black arrow) was defined as the minimal DNA fragment affected by any imbalance in the majority of cases. Correspondence to *SIRPB1* gene was found (red arrow). Please note the occurrence of other abnormalities rather than amplification within the MAR or in the adjacent area in some cases.

Importantly, among all the 20p13 lesions occurring in our PMF patients, the amplification reported in 11/20 patients identified a minimally affected region (MAR). Specifically the MAR was a segment of DNA amplified 4 times (average copy number = 4,1) on chromosome 20, extending for about 9,911 bps from 1572019 to 1581930 nucleotides and overlapping the *SIRPB1* gene locus (Figure 5).

### TaqMan Copy Number assay of MAR

In order to validate the results obtained with the SNP-A, we used the TaqMan Copy Number Assay using a specific probe overlapping under the CNV of interest on 20p13 cytoband (Hs04039030_cn; Applied Biosystems; Foster City, CA). We studied 10 samples, in which the MAR appeared to be involved at SNP-A analysis, and for which residual DNA was available. Furthermore, we analyzed 6 additional DNA samples, including 1 negative control as well as DNA from matched non neoplastic T lymphocytes of 5/10 patients. We also tested the target copy number amplification on a human genomic DNA control (Reference Genomic DNA, 103, Affymetrix, Santa Clara, CA, USA). The analysis was performed in quadruplicate and three times in three different days for further control.

**Figure 6:**
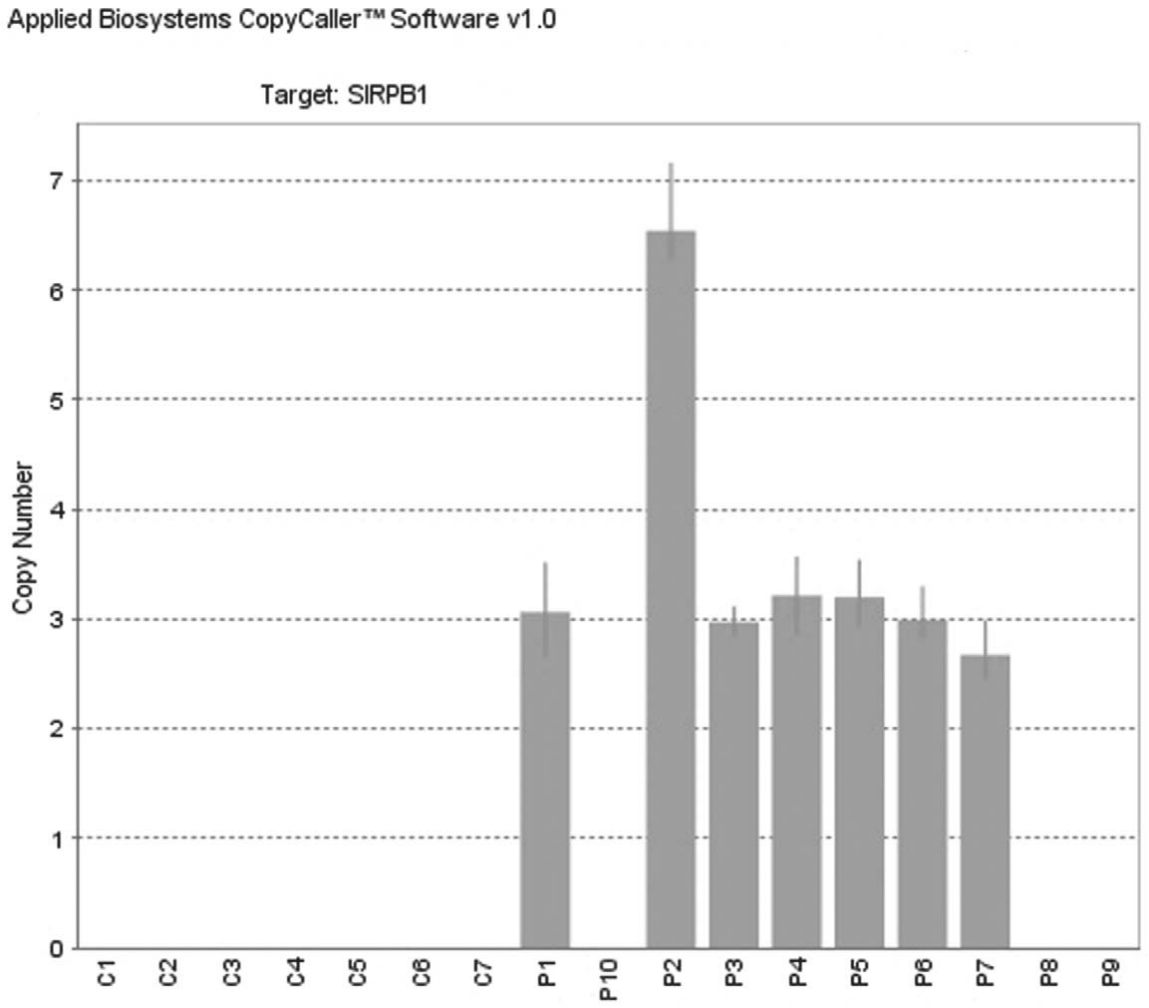
Validation of MAR abnormalities TaqMan Copy Number Assay. Each column represents a single genome (P = patient; C = control). Bars correspond to standard deviations. On the y axis, detected copy number values are presented. C1 = gDNA from Affymetrix; C2 = healthy donor; C3–C7 = patients matched DNA from non neoplastic cells; P1–P10 = patients.

High inter-assay and intra-assay reproducibility was recorded. In particular, analogue results were obtained in the replicates and consistent results were found within each repetition with a σΔCt below 0,15. Overall, the assay confirmed the SNP-A results, with a consistency of 70%. Specifically, we detected the target copy number to be amplified in 7/10 patients (Figure 6).

### SIRPB1 protein detection

**Figure 7:**
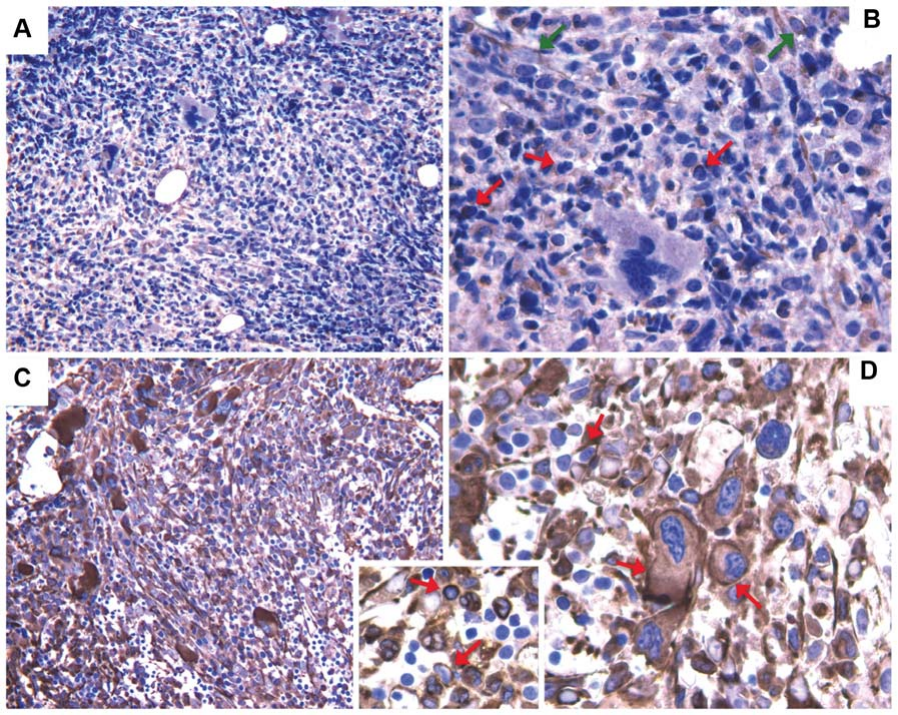
Immunhistochemical expression of SIRPB1. A–B) In PMF cases not showing 20p13 amplification, SIRPB1 expression is mainly confined to BM macrophages and dendritic cells (B, green arrows) and to scattered myeloid cells (B, red arrows). C–D) By contrast, in cases with 20p13 amplification, SIRPB1 is diffusely expressed by myeloid and megakaryocytic elements with a combined cytoplasmatic and surface expression (D, inset, red arrows). Images are relative to two representative cases out of ten tested (5 per condition). Anti-SIRPB1 immunostaining with the Strept-ABC method and DAB chromogen (brown signal). Original magnifications: a, c ×200; b, d, inset ×400.

Following the detection of a 20p13 amplification overlapping the *SIRPB1* gene, we tested the expression of SIRPB1 protein by immunohistochemistry on 5 PMF cases characterized by 20p13 amplification and on 5 PMF cases not showing the molecular abnormality. Interestingly, we found a different expression of SIRPB1 between the two groups (Figure 7). In particular, the protein was widely expressed in all myeloid lineages, including megakaryocytes, macrophages, dendritic cells, and myeloid precursors in cases with *SIRPB1* amplification. On the contrary, it was essentially restricted to macrophages and dendritic cells in PMF cases not carrying 20p13 abnormalities (Figure 7). Of note, in cases characterized by 20p13 amplification, SIRPB1 expression showed a combined intracytoplasmatic and membranous pattern, while it was mainly bound to the membranes in the other cases (Figure 7).

## Discussion

In this study, we aimed to identify novel cytogenetic abnormalities in PMF patients by using high-density SNP-A. Previous studies showed the ability of such technique in detecting novel genetic lesions in both myeloid and lymphoid disorders [24]–[26]. In particular, SNP-A profiling retains several advantages if compared to metaphase cytogenetic (MC), including the ability to detect lesions even when cells are not actively dividing, a condition favourable to the investigation of chronic myeloid neoplasms (CMN), and definitely higher sensitivity and capacity to detect aUPD. Noteworthy, the latter two features also distinguish SNP-A analysis from CGH. On the other hand, a major limitation of SNP-A, in comparison to MC, is its inability to recognize chromosomal translocations [27].

Indeed, with this approach we were able to detect a remarkable number of lesions (2,765 CNVs) including aUPD, deletion and amplification, many of which (992/2,765; 36%) turned out to be previously uncovered. Notably, according to our study design, all copy number changes detected by SNP-A paired analysis in the training set, were then validated in a test set. Specifically, the correspondence between the two groups was significant (p = 0.0053), with 44% of lesions confirmed in both (Figure S2).

Concerning the lesions previously associated with MPN, we identified del(13q), del(20q), and abnormalities on chromosome 1, +9 +8 and del(5q). Actually, we identified the presence of different regions of aUPD on chr9 at *JAK2* locus [16], on chr 13q, on chr 1 and on chr 11 [17], [28].

In particular, we observed large regions of aUPDs in correspondence to the *MPL*, *CBL and RB1* genes which are commonly mutated or deleted in PMF.

Importantly, as internal validation, we also recognized two lesions previously detected by MC in our cohort (namely del(5q) and trisomy 8). Thus, SPN-A karyotyping was confirmed to be an effective tool for the identification of both cryptic and larger genomic abnormalities [5], [29]. Taken together, our results confirmed the presence of a complex and heterogeneous karyotype in PMF patients, basically consistent with previous studies [7] and proposed novel CNVs for further characterisation.

As far as newly identified aberrations were concerned, we focused on the lesions which recurred at higher frequency in our series, thus being candidate to retain a potential pathogenic significance. Interestingly, as indicated by other studies using less sensitive techniques [30], PMF was confirmed to have heterogeneous lesions at cytogenetic level. Actually, all patients presented with a complex karyotype, with a minimum number of CNV/patient of 188. Remarkably, however, when a paired analysis was performed in the training set, no lesion was consistently present in all the cases, not even in up to 60% of them. On the other hand, a few new lesions were found in 50% of patients. Among them, we focused on an amplification affecting the cytoband 20p13, since it proved to be the most frequent in the entire series (training + test set), the overall prevalence of such CNV being 55%. Noteworthy, if confirmed in larger series and functionally relevant, it would represent the commonest genomic lesion so far described in PMF, together with *JAK2* somatic mutations.

To better understand the context of this lesion we primarily extend our attention to the adjacent areas, in the 20p13 cytoband. Interestingly, we found that all the patients except one (19/20), have at least one alteration (amplification, deletion or aUPD) occurring in the 20p13 cytoband. Subsequently, we assessed the prevalence of 20p13 abnormalities in an independent validation data set. Specifically, we studied a group of 60 myeloid tumours, basing on previously published data which were available online. Remarkably, we confirmed the presence of lesions affecting 20p13 cytoband in 7/14 MDS/MPN patients, 5/7 PMF patients and 1/39 MPN patient. Of note, this finding was then consistent with the concept that different myeloid malignancies may share common genetic abnormalities [31], [32]. In this regard, 20p13 gains were recently described in MDS [22], though occurring at very low frequency. Nevertheless, other loci on chromosome 20 have been found to be involved in myeloid malignancies, though the implicated genes and the exact consequences are poorly known [21], [23].

Importantly, among all the 20p13 lesions detected in our PMF patients, we could define the CNV occurring with 55% of frequency as a minimally affected region (MAR). In particular the MAR is an amplification spanning over 9,911 bps, in correspondence to the *SIRPB1* gene locus.

Subsequently, we investigated whether the copy number amplification within the *SIRPB1* gene could be eventually reflected at protein level. SIRPB1 is a member of the signal-regulatory-protein (SIRP) family, belonging to the immunoglobulin superfamily. SIRPB1 is expressed in neurons and a limited subset of hematopoietc cells mainly consisting of macrophages and dendritic cells (DCs) [33]–[36]. Importantly, the efficient cell-surface expression of SIRPB1 requires the association with DAP12, a dimeric adaptor ITAM- bearing protein, located in the plasma-membrane. Upon stimulation, the SIRPB1/DAP12 complex induces tyrosine phosphorylation of a number of kinases including the mitogen-activated protein kinases (MAPKs), ERK1, and ERK2. In addition, it binds the tyrosine kinase SYK, leading to the activation of the SYK-JAK-STAT intracellular cascade [37]–[40].

To date no involvment of SIRPB1 protein has been reported in any diseases, except for the Alzheimer's disease where its up regulation is responsible to increase the phagocytosis process [41]. However, 20p13 abnormalities (amplifications, deletions or translocations) have been reported in sporadic cases of osteosarcoma, adenocarcinoma, Adult T-cell lymphoma/leukemia (HTLV-1+), and chronic myeloid leukemia according to the Cancer Chromosome database (http://www.ncbi.nlm.nih.gov/cancerchromosomes).

Indeed, in our series we found SIRPB1 significantly more expressed in patients carrying the genomic abnormality rather than in the other cases. Specifically, in patients not harboring the 20p13 amplification, we found, as attended, a sporadic expression of SIRPB1 mainly restricted to dendritic cells (DCs) and macrophages. Conversely, in patients carrying the 20p13 alteration, we appreciated an intense expression of SIRPB1 in all myeloid cells, this reflecting the occurrence of the lesion in a common myeloid progenitor. Furthermore, of note, in case of 20p13 amplification SIRPB1 expression was not limited to the plasma-membrane, but was extended to the cytoplasm as well. It is conceivable that this phenomenon reflected the complete saturation of the SIRPB1 binding sites on the plasma-membrane leading to an abnormal accumulation in the cytoplasm. Bearing in mind the involvement of SIRPB1 in SYK-JAK-STAT signalling, it is possible that a deregulated SIRPB1 expression and/or function might eventually sustain proliferation and survival of affected cells. Intriguingly it may represent an alternative stimulation in JAK2^wt^ cases, possibly having an additive/synergistic effect in JAK2^mut^ ones. In this regard, the two lesions did not appear to be mutually exclusive, as 20p13 amplification was recorded in both JAK2^wt^ and JAK2^mut^ patients.

In conclusion, we detected a novel recurrent lesion involving 20p13 and *SIRPB1* gene in PMF patients. Future studies are definitely warranted in order to further characterize the genomic lesion and its functional consequences, as well as to assess the possible clinical relevance of such lesion.

## Supporting Information

Figure S1 **Distribution of CNVs across patients.** On the y-axis, the CNV number for each patient is presented. Amplifications (both with and without LOH) in blue, acquired uniparental disomy regions (aUPD) in green and deletions (both with and without LOH) in red.
(TIF)

Figure S2 **CNVs recorded in the test set.** A) Karyoview of test set: Amplification with LOH (Light blue), Amplification without LOH (Dark blue), Copy Neutral LOH (aUPD, Green), Deletion with LOH (Violet), and Deletion without LOH (Cyclamen) are represented. All the 2,096 lesions recorded in the test set are depicted. B) Venn-Diagram representing the significant overlap between the cytobands recognised as affected by any imbalance in the training (violet) and test (blue) set, respectively (p = 0.0053).
(TIF)

Figure S3 **Acquired uniparental disomy (aUPD) in PMF patients.** Amplification with LOH (Light blue), Amplification without LOH (Dark blue), Copy Neutral LOH (aUPD, Green), Deletion with LOH (Violet), and Deletion without LOH (Cyclamen) are represented. Please note each row represents a single patient and in particular empty rows represent different cases randomly selected as negative controls. A) Three patients reported aUPD regions (green bars) in correspondence to the *CBL* gene. B) Three patients presented aUPD regions (green bars), one patient reported a deletion with LOH (violet bar) one patient showed a deletion without LOH (cyclamen bar) and two patients presented a micro amplification without LOH (blue bars): all these alterations overlapped the *RB1* gene. C) Two patients reported aUPD regions (green bars) in correspondence to the *MPL* gene.
(TIF)

Figure S4 **Recurrence of CNVs across PMF patients.** On the x-axis, the number of patients (evaluated in the training set) is presented. On the y-axis, the number of CNVs recurring in a certain number of patients is offered.
(TIF)

Figure S5 **Recurrence of 20p13 in a panel of myeloproliferative neoplasms.** Basing on *in silico* analysis, 20p13 abnormalities were recorded in a significant proportion of MPN cases. On the x-axis, three different datasets are reported, based on different microarray technology (Affymetrix SNPs-array 6.0, Affymetrix Human Mapping 250K Nsp, and Affymetrix Human Mapping 50K Array Xba 240, respectively).
(TIF)

File S1 **All the 2,765 CNVs detected in the training set patients are reported. For each lesion is indicated the length, the chromosome , the cytoband and the genes eventually involved.**
(XLS)

Table S1 **Patients Characterisctics.**
(DOC)
