# Supplementary figures and images for: SNPs Array Karyotyping Reveals a Novel Recurrent 20p13 Amplification in Primary Myelofibrosis
Source: PLoS One. 2011 Nov 14;6(11):e27560. doi: 10.1371/journal.pone.0027560 (PMC3215741; doi:10.1371/journal.pone.0027560)

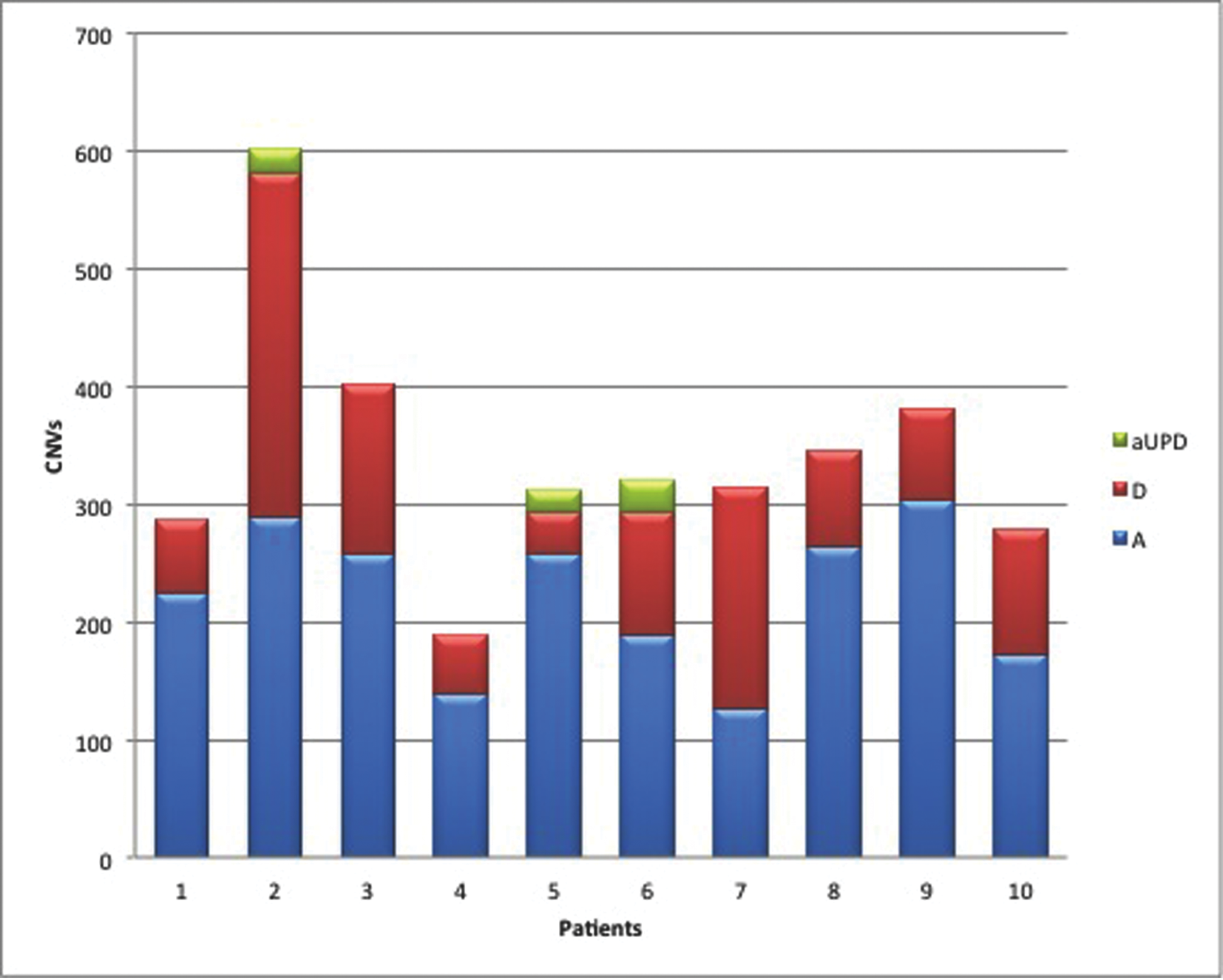

Supplement: Figure S1 — Distribution of CNVs across patients. On the y-axis, the CNV number for each patient is presented. Amplifications (both with and without LOH) in blue, acquired uniparental disomy regions (aUPD) in green and deletions (both with and without LOH) in red. (TIF) [file pone.0027560.s001.tif]

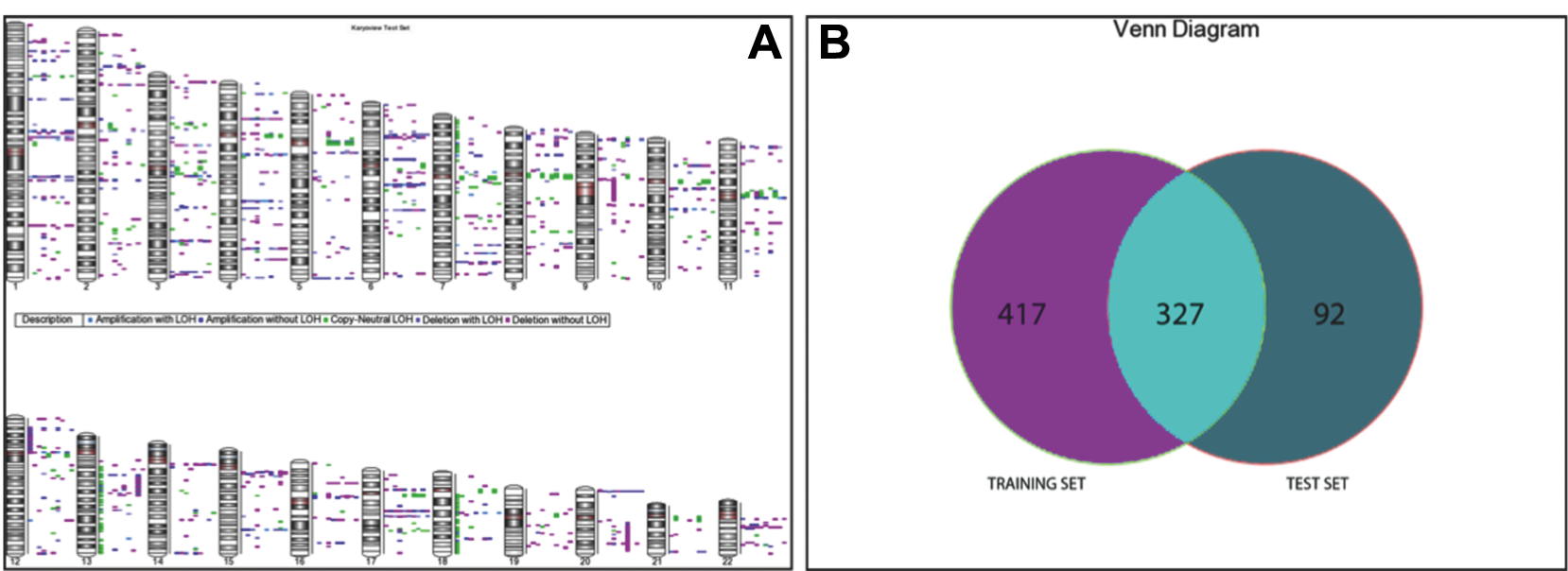

Supplement: Figure S2 — CNVs recorded in the test set. A) Karyoview of test set: Amplification with LOH (Light blue), Amplification without LOH (Dark blue), Copy Neutral LOH (aUPD, Green), Deletion with LOH (Violet), and Deletion without LOH (Cyclamen) are represented. All the 2,096 lesions recorded in the test set are depicted. B) Venn-Diagram representing the significant overlap between the cytobands recognised as affected by any imbalance in the training (violet) and test (blue) set, respectively (p = 0.0053). (TIF) [file pone.0027560.s002.tif]

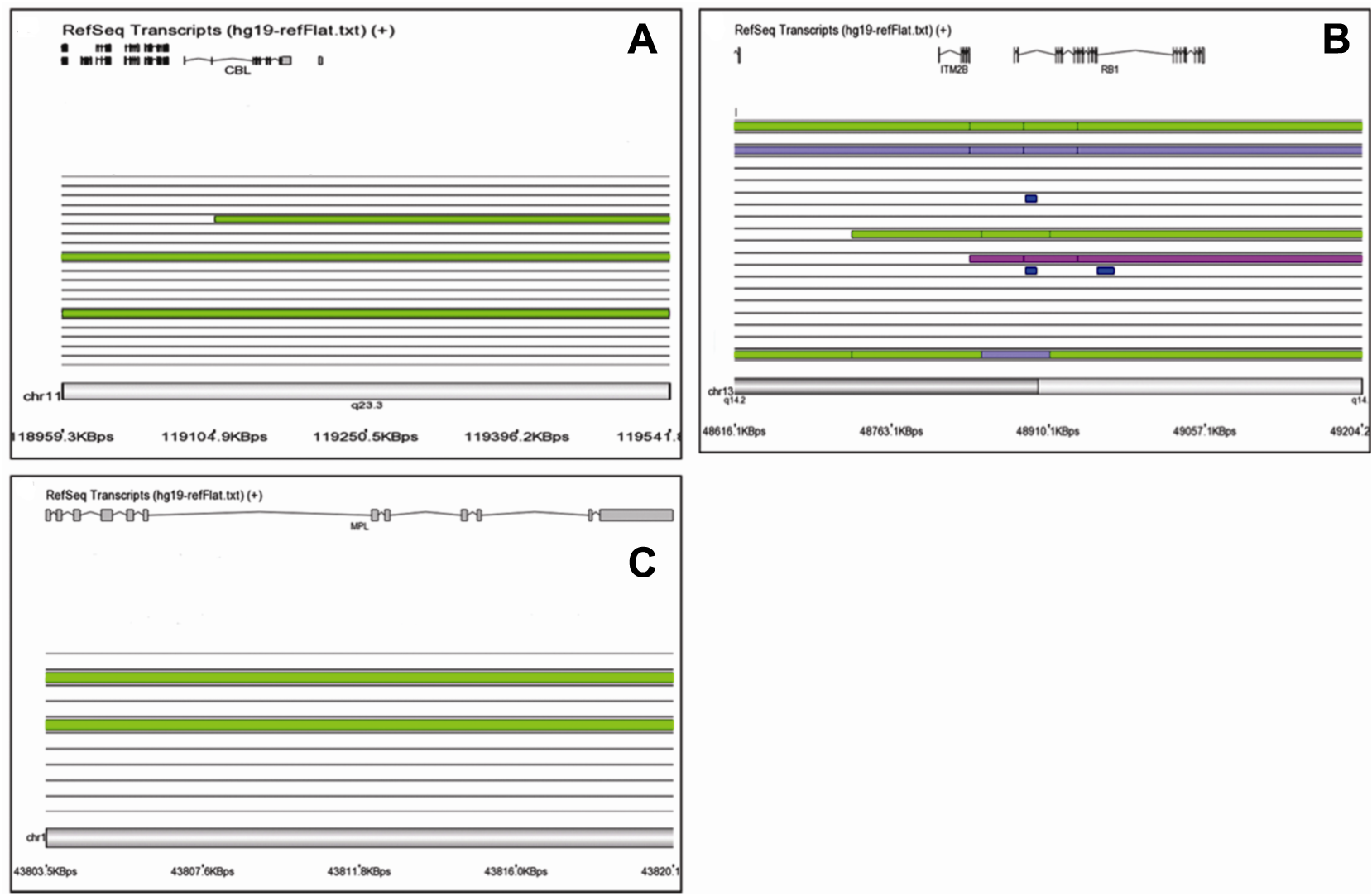

Supplement: Figure S3 — Acquired uniparental disomy (aUPD) in PMF patients. Amplification with LOH (Light blue), Amplification without LOH (Dark blue), Copy Neutral LOH (aUPD, Green), Deletion with LOH (Violet), and Deletion without LOH (Cyclamen) are represented. Please note each row represents a single patient and in particular empty rows represent different cases randomly selected as negative controls. A) Three patients reported aUPD regions (green bars) in correspondence to the CBL gene. B) Three patients presented aUPD regions (green bars), one patient reported a deletion with LOH (violet bar) one patient showed a deletion without LOH (cyclamen bar) and two patients presented a micro amplification without LOH (blue bars): all these alterations overlapped the RB1 gene. C) Two patients reported aUPD regions (green bars) in correspondence to the MPL gene. (TIF) [file pone.0027560.s003.tif]

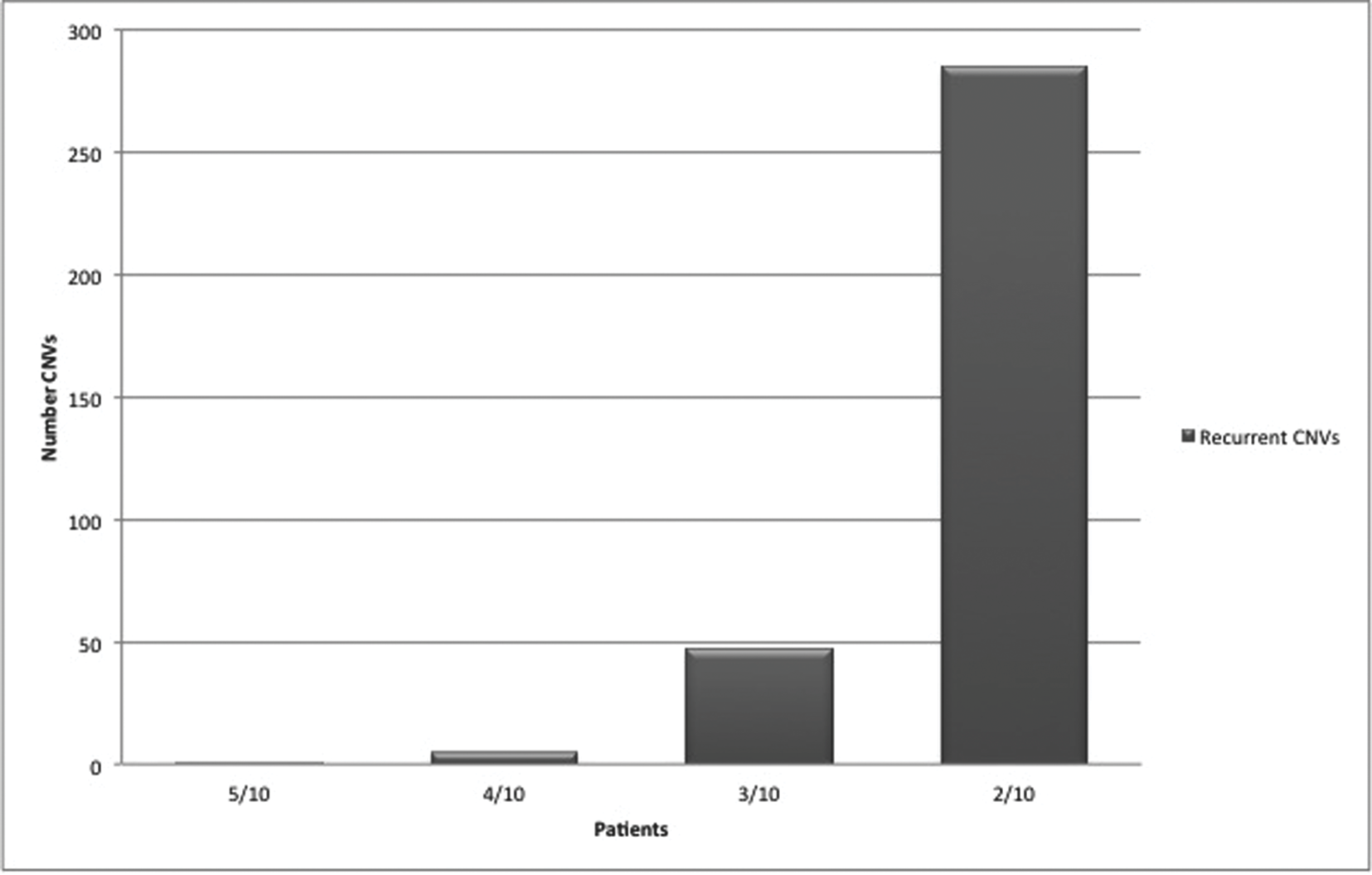

Supplement: Figure S4 — Recurrence of CNVs across PMF patients. On the x-axis, the number of patients (evaluated in the training set) is presented. On the y-axis, the number of CNVs recurring in a certain number of patients is offered. (TIF) [file pone.0027560.s004.tif]

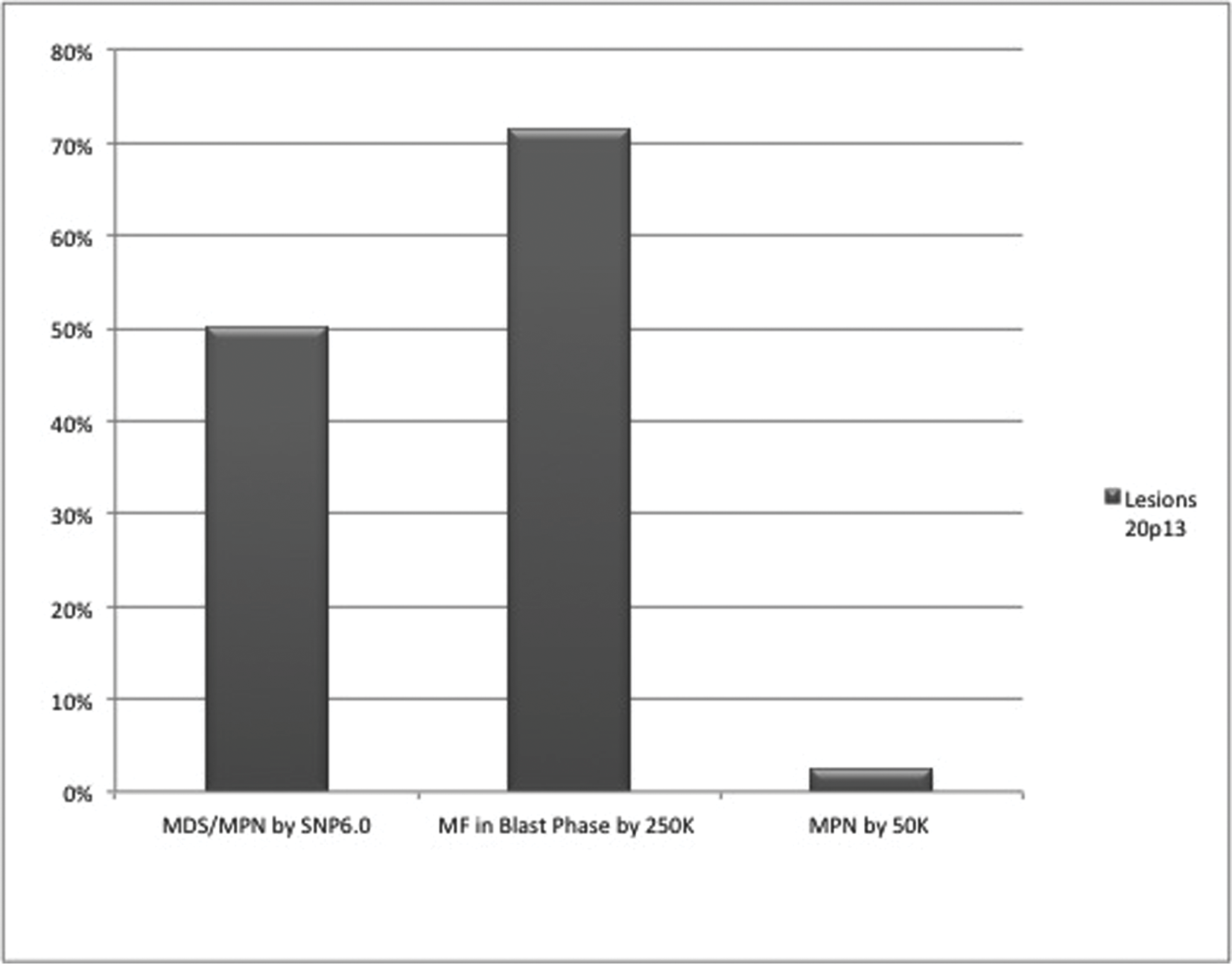

Supplement: Figure S5 — Recurrence of 20p13 in a panel of myeloproliferative neoplasms. Basing on in silico analysis, 20p13 abnormalities were recorded in a significant proportion of MPN cases. On the x-axis, three different datasets are reported, based on different microarray technology (Affymetrix SNPs-array 6.0, Affymetrix Human Mapping 250K Nsp, and Affymetrix Human Mapping 50K Array Xba 240, respectively). (TIF) [file pone.0027560.s005.tif]
