# Supplementary material for: SNPs Array Karyotyping Reveals a Novel Recurrent 20p13 Amplification in Primary Myelofibrosis
Source: PLoS One. 2011 Nov 14;6(11):e27560. doi: 10.1371/journal.pone.0027560 (PMC3215741; doi:10.1371/journal.pone.0027560)
Supplement: Table S1 — Patients Characterisctics. (DOC) [file pone.0027560.s007.doc]

**Table 1.** Patients’ characteristics

|  | **Training set** | **Test set** |
| --- | --- | --- |
| Patients’ number | 10 | 10 |
| Median age, years (range) | 68 (53-82) | 74 (44-81) |
| Patients aged over 65 | 5 (50%) | 8 (80%) |
| Female/male ratio | 5/5 (50%) | 4/6 (40%) |
| JAK2^wt^ /JAK2^mut^ | 6/4 (60%) | 4/6 (40%) |
| Median follow up, months (range) | 24,5 (7-96) | 38,5 (8-120) |
| Leukaemic transformation | 0 (0%) | 2 (20%) |
